# Providing care at a most vulnerable time

**DOI:** 10.1093/crocol/otag030

**Published:** 2026-04-11

**Authors:** Paula Prieto Tizzard, Shervin Rabizadeh, Joel R Rosh

**Affiliations:** Phoenix Children’s Hospital, Phoenix, AZ, USA; Department of Pediatrics, Cedars-Sinai Guerin Children’s, Cedars-Sinai F. Widjaja Inflammatory Bowel Disease Institute, Cedars-Sinai Health Sciences University, Los Angeles, CA, USA; Northwell Health, Cohen Children’s Medical Center, Zucker School of Medicine, New Hyde Park, NY, USA

Successful management of inflammatory bowel disease (IBD) requires navigation of medical as well as psychosocial challenges. In addition to the potential of disease-related complications such as hospitalization and surgery, those affected by IBD can face significant financial distress that is disproportionately impactful on individuals who are less than 65 years of age as well those who have a lower income.^1^ Since IBD is commonly diagnosed during adolescence and young adulthood, the period of transition of care from a pediatric to an adult clinical team poses a compounded risk for financial stress and an interruption in healthcare.^2^

Young adulthood is often a vulnerable time characterized by major life changes such as starting college or entering the workforce, moving away from home, transitioning from parental to self-insurance, and the overall development of self-independence.^3^ The manuscript by Maltz et al. in this edition of Crohn’s and Colitis 360, brings to attention some of the difficulties that young adult patients may endure compared to other age groups of patients affected by IBD.^4^ The young adults studied were more likely to experience financial challenges in paying IBD related costs compared to other groups. They were more likely to work an extra job or work more hours to pay IBD related medical bills. Further, young adult patients were more likely to experience step therapy mandates and have less knowledge on the questions to ask if experiencing insurance coverage issues.

Transition of care for adolescents and young adults has been a hot topic for some time. Models for successful transition include dedicated transition clinics^5^ but this is not universally practical nor is there a clear structure for optimization of such efforts. The biology of IBD in adolescents and young adults complicates these issues. The phenotypes of IBD in this age group have been shown to be more clinically aggressive with higher rates of perianal and upper GI involvement in Crohn’s disease (CD); and higher rates of pancolitis in ulcerative colitis (UC).^6–9^ These unique disease characteristics as well as the psychosocial needs of young patients with IBD highlight the need for holistic and comprehensive care, especially during the time of transition.

This time of increased need is challenged by substantially higher rates of gaps in insurance coverage and other financial barriers compared to both younger children and older adults with IBD. Before the Affordable Care Act’s (ACA) dependent coverage provision, the 19-25 year old demographic represented the highest proportion of uninsured IBD patients in the US. Following the ACA provision allowing young adults to remain on parental insurance through age 25, uninsured rates decreased from 14.1% in 2010 to 10.1% in 2011.^10^ Despite this improvement, young adults continue to experience disproportionate insurance-related challenges. Loss of insurance coverage during transfer to adult care may be exacerbated by aging out of parental coverage or children’s Medicaid/CHIP eligibility.^11^

Financial consequences are significant. Young adults with IBD experience higher rates of difficulty paying medical bills, with 21% reporting such struggles. Those with lower income and education levels face even greater challenges, with household income below $60 000 being associated with 3.7-fold increase in odds for financial toxicity.^1^ This is consistent with the troubling finding of Maltz et al. that young IBD patients often need to seek additional income sources to pay for medical related costs.

Even for those who have insurance, restrictions within plans can be a major obstacle. Prior authorizations, coverage denials, step-therapy requirements, and other administrative barriers frequently delay care and contribute to worse health outcomes. Combined with the challenges of transitions in care, these delays have the potential to lead to gaps in routine care, disease related complications, higher healthcare costs, and ultimately, reduced quality of life.^6^

The transition process is complex and requires a well-coordinated effort. Parents, patients, pediatric and adult IBD healthcare teams, and insurance companies all play key roles. Young adults with IBD are particularly susceptible to issues of healthcare access including lapses in care, logistical challenges, and financial toxicity as they navigate the healthcare system. It is important to address the financial landmines and potential medical coverage pitfalls that face adolescents and young adults. Having these discussions as a part of clinical visits will enhance shared decision-making and lead to proactive planning of preventative strategies. This seems like a great first step while the findings of Maltz et al. hopefully lead to increased educational resources and intensified advocacy that will drive positive systemic change.
